# Case of Empyema

**Published:** 1834-01-01

**Authors:** John Bullock

**Affiliations:** Apothecary, Westminster Hospital.


*To the Editor of the Medical Quarterly Review.*

SIR: Having read in the first number of your Quarterly Journal an interesting case of Empyema, treated by Dr. STROUD, I thought the following case, terminating more fortunately, might prove worthy of notice to some of your readers.

Yours, respectfully,

JOHN BULLOCK,
*Apothecary, Westminster Hospital.*

*December* 23, 1833.

Silas Hann, a tailor, æt. twenty, of dark complexion, and weak habit of body, was admitted August 6th, 1833, under the care of Dr. ROE, at the Westminster Hospital.

About six weeks previous he enjoyed his usual health, (which was never very good, having for some years been subject to a dry, hacking cough,) when he was seized with pain in the limbs, and fatigue on slight exertion. After a few days he experienced a sharp lancinating pain under the right breast, shooting to the shoulder, and increased by inspiration, with dry cough. He was relieved at the time by some medicine and a blister. July 31st, after exposure to the night air, he became worse: at present feels very weak, has a dry cough, more troublesome at night; breathing short and hurried; pain in the right side increased on inspiration and pressure in the intercostal spaces; generally reclines on the right side; pulse 120, weak; tongue reddish, brown in the centre, white at the edges; is fatigued by sitting up. On percussion, there is extreme dullness of sound over the right side of the thorax, before and behind, although it diminishes somewhat above the sixth rib. Absence of respiratory murmur generally over the same side, except in one or two places, where it was indistinctly audible. Ægophony at the inferior angle of scapula. The liver descends considerably lower than usual. Respiratory murmur of the left side strong and puerile. On the introduction of a grooved needle between the sixth and seventh ribs, a clear straw-coloured fluid escaped.

7th. Two P.M. A trocar was introduced at the same point, and six pints of fluid, interspersed with numerous flocculi of lymph, drawn off; a quantity of air at the same moment rushing into the pleural cavity, through the instrument. The wound was accurately closed, and he expressed himself relieved.—Calomel. Pulv. Opii, *aa* gr. i. statim et h.s.

Ten P.M. Very weak, but not more so than before the operation. Pulse 108, fuller. Tympanitic sound on percussion, with absence of respiratory murmur.

8th August. Passed a tolerably good night. He coughs less, and breathes more freely. Pulse 116, full, and strong; bowels not open.—Ol. Ricini, ℥ss. statim. ℞. Acid. Hydrocy. dil. m. xij.; Tinct. Scillæ, ʒiss.; Aq. distill. ℥viij. ℥i. secundâ quâque horâ. Empl. Canth. lateri dextro. Rep. Pil. Calom. et Opii, h. s.

August 9th. Some anxiety of countenance; pulse 100; skin dry and hot. Adde Mist. Vin. Colchici, m. xl. Tongue brown; bowels open; slept well, but sweated much in the early part of the night.

August 10th. Less anxiety of countenance; slept well; pulse 116.

August 11th. Did not sleep so well. A full inspiration excites cough and pain in the side. Pulse 124, full.—Hirudines xviij. lat. appl.

August 12th. Was much relieved by the leeches. Pulse 120.—Rep. mist.

August 15th. Respiration audible on the fore and upper part of the right side of the thorax. Pulse 116; very little cough.

August 17th. Feels very low: pulse 112.—Omitt. mist. Pulv. Opii, gr. iss. h. s.

August 19th. Slept well; still very weak; pulse 108.—℞. Inf. Gent. c. ℥vijss.; Tinct. Rhei, ℥ss.; Conf. Arom. ʒi.; Cretæ pp. ʒij. ℥i. ter die.

August 21st. Very weak; pulse 104; skin rather hot; slept well.—Omitt. Mist. Gent. Vini Lusitan. ℥iv. quotidie. ℞. Sulph. Quin. gr. xvj.; Acid. Sulph. d. ʒi.; Mist. Camph. ℥viij. ℥i. ter die.

August 23d. Looks more emaciated and feeble; pulse 110; tongue dry and reddish.

August 24th. A hollow sharp-pointed instrument, about the length of, and somewhat larger in diameter, than a common darning needle, with a hole about an inch from the extremity, accurately adapted to a stomach-pump, was introduced into the right side, and the air exhausted. He could draw a deep breath with more ease, there was no tympanitic sound, and the respiratory murmur became much more audible, immediately after the operation.—Omit the medicine.

August 25th. Breathes with more freedom; pulse 112, weak; tongue dry and chapped.—Continue the wine.

August 27th. Dullness of sound on percussion has much increased; pulse 104.

August 31st. Dullness on percussion increasing; pulse 106.

September 2d. Cough rather more troublesome; respiration not heard over so great an extent; pulse 112, weak; tongue moist.

Sept. 4th. Pulse 104; appetite improving; respiration heard above the fourth rib anteriorly and posteriorly at the root of the lungs, and hollow sound on percussion over the same space.—A mixture containing Acet. Potass. ter die.

Sept. 11th. Feels stronger; coughs less. Right side of the thorax manifestly contracted; the shoulder much lower than on the left; in fact, exhibiting precisely the appearance of plates 6 and 7 in Laennec's work.

He continued much the same up to the 25th, when a blister was applied, in consequence of recurrence of cough and pain.

Sept. 30th. Neither cough nor pain; pulse 116; tongue clean; percussion elicited a clearer sound on the upper and fore part of the right side.—Pil. Sapon. c̄ Opio, gr. v. o. n.

October 9th. Continues to improve daily.

Oct. 19th. Respiration is heard quite distinct and natural at the upper third of the right side; not so below. Sound on percussion clear superiorly, dull inferiorly.—℞. Sulph. Quin. gr. xvi.; Sulph. Ferri, gr. xij.; Acid. Sulph. d. ʒss.; Mist. Camph. ℥viij. ℥j. ter die.

Oct. 26th. Respiration gradually extending over the whole of the right side.

November 16th. Dismissed cured.

